# Medication adherence and blood pressure control in treated hypertensive patients: first follow-up findings from the PREDIcT-HTN study in Northern Bangladesh

**DOI:** 10.1186/s12889-025-21409-z

**Published:** 2025-01-21

**Authors:** Ahmed Hossain, Gias Uddin Ahsan, Mohammad Zakir Hossain, Mohammad Anwar Hossain, Probal Sutradhar, Sarowar-E. Alam, Zeeba Zahra Sultana, Heba Hijazi, Syed Azizur Rahman, Mohamad Alameddine

**Affiliations:** 1 College of Health Sciences, University of Sharjah, Sharjah, United Arab Emirates; 2 Department of Public Health, North South University, Dhaka, 1229 Bangladesh; 3 Canadian University of Bangladesh, Dhaka, Bangladesh; 4 Rangpur Hypertension and Research Center, Rangpur, Bangladesh; 5 Department of Health Management and Policy, Faculty of Medicine, Jordan University of Science and Technology, Irbid, 22110 Jordan

**Keywords:** Hypertension, Non-communicable disease, Medication adherence, Hypertension management, Bangladesh

## Abstract

**Introduction:**

Adherence to antihypertensive medication is crucial to control blood pressure (BP) and hypertension management outcomes. In Bangladesh, as in many other countries, poor adherence to medication represents a challenge to effective hypertension management. This study aims to investigate the prevalence and relationship between medication adherence and BP management among hypertensive patients in Bangladesh.

**Methods:**

The PREDIcT-HTN study in Northern Bangladesh aims to evaluate major adverse clinical events in treated hypertensive patients. The study involves 2643 hypertensive patients from a medical center, with data collected through baseline information and yearly follow-ups until 2025. The first follow-up visit was conducted between January and March 2021. Following the 2020 ISH-global hypertension guideline, patients were classified as having controlled BP, grade-I, or grade-II uncontrolled BP. Patients were divided into three groups (good, moderate, and poor) based on their 9-item Hill-Bone medication adherence scale. A multinomial regression analysis was conducted to identify the association between medication adherence and BP control after adjusting potential confounders.

**Results:**

Analysis of 2276 hypertensive patients (mean age 51.31 ± 11.58 years) revealed that 36.1% had grade-I and 24.2% had grade-II uncontrolled hypertension. Most patients (78%) displayed moderate adherence, and 15% showed poor medication adherence. Certain patient subgroups had higher rates of poor adherence: females (17.1%) compared to males (12.2%), rural residents (22.4%) compared to city-dwellers (12.2%), and newly diagnosed patients (17.2%) compared to those diagnosed 2–5 years earlier (12.6%). Multivariable analysis found a strong association between medication adherence and BP control. Compared to poor adherence, moderate adherence (relative risk ratio (RRR):0.50, 95%CI:0.36–0.68) and good adherence (RRR:0.56, 95%CI:0.35–0.91) were associated with better control. Increasing age, rural living, and uncontrolled hypertension were also linked. Comorbidities worsened BP control, and managing multiple medications contributed to poor adherence and grade-II hypertension in patients.

**Conclusion:**

The high prevalence of uncontrolled hypertension in Bangladesh underscores the need for improved treatment strategies. Addressing medication adherence is essential for better BP control, with particular attention needed for women, rural residents, and newly diagnosed individuals. A comprehensive approach is warranted, including strategies to enhance adherence, early diagnosis, personalized treatment, and simplified medication regimens. These efforts align with the UN's 2030 SDGs, emphasizing targeted interventions for equitable healthcare access and outcomes.

**Supplementary Information:**

The online version contains supplementary material available at 10.1186/s12889-025-21409-z.

## Introduction

Hypertension, a prevalent global disease, significantly increases the risk of chronic conditions like heart disease, stroke, and kidney problems [1, 2]. It also causes substantial mortality among adult population and identified as the third leading cause of deaths worldwide [2]. Moreover, about half of the deaths caused by cardiovascular diseases are directly linked to hypertension [1]. The Low- and Middle-Income Countries (LMICs), particularly from South Asian region is going under an epidemiological shift and have started to experience a steep rise in the cases of Non-Communicable Disease (NCD) including hypertension. Bangladesh shares a similar scenario with a current prevalence of 25.2% hypertension among its adult population [3–5].

Despite the high burden, only one-third of the hypertensive population of LMICs are aware of their condition, and only around 8% have their blood pressure under control [4, 6]. According to studies, the prevalence of uncontrolled hypertension in Bangladesh ranges from 25 to 50% [4, 7, 8]. Due to the chronic nature of the disease and indefinite treatment duration, adherence to treatment remains a significant challenge among the hypertensive patients [9]. The World Health Organization (WHO) declared poor adherence to medication as the most significant cause of uncontrolled blood pressure (BP) and reported that about 50–70% of people fail to take their antihypertensive medication as prescribed [10]. Likewise, International Society of Hypertension reported that non-adherence affects 10–80% of patients with hypertension, leading to their poor prognosis [11]. Treatment adherence is also considered a vital way of preventing negative consequences of chronic diseases and ensuring overall success of the treatment [12, 13]. To facilitate the adherence to treatment, hypertensive patients are required to attend follow-up visits at healthcare centers at regular interval [14, 15].

Studies found that hypertensive patients with regular follow-up history have succeed to attain a better prognosis of their disease [16]. On the other hand, patients who fail to follow up with their healthcare providers are more likely to develop uncontrolled BP or medication side effects, which may have been avoided if they had attended follow-up sessions. Furthermore, the presence of comorbid conditions in addition to hypertension is thought to complicate the treatment procedures and influence a poor prognosis among hypertensive patients [17].

While there is growing evidence on the importance of medication adherence, follow-up history, and status of multimorbidity in the comprehensive management of hypertension, Bangladesh has not yet addressed or incorporated these issues in the national guideline for management of hypertension [18]. Bangladesh government, however, has shown its commitment by adopting a multi-sectoral action plan for NCD control and prevention that will run from 2018 to 2025 [19]. The strategy aims to improve population health by implementing evidence-based policies and programs.

As hypertension is not only one of the leading NCDs, but also a risk factor for many chronic diseases, a comprehensive hypertension management strategy will be vital to the successful implementation of this multi-sectoral plan. There is a dearth of information regarding the role of medication adherence, regular follow-up, and comorbid status in the management of hypertensive patients in the context of Bangladesh. The available data is not sufficient to draw inferences regarding the distribution of hypertension type and trends in anti-hypertensive medication adherence across BMI, number of medications, follow-up, multimorbidity, and socio-demographic factors in Bangladesh. The study seeks to determine the prevalence of hypertension type and factors associated with uncontrolled hypertension in treated hypertensive patients in Bangladesh, focusing on unraveling the intricate relationship between medication adherence and BP control. By evaluating socio-demographic and clinical factors, such as age, gender, education, and comorbid conditions, the study seeks to identify patterns of adherence and their impact on hypertension management. Finally, the study will generate evidence to strengthen the national hypertension management strategy and ensure optimal clinical benefits for patients.

## Methods

### The PREDIcT-HTN study

The PREDIcT-HTN study is a longitudinal investigation spanning 5 years, aiming to explore uncontrolled hypertension and adverse cardiovascular events in treated hypertensive patients residing in Northern Bangladesh. The study is conducted at the Hypertension and Research Centre (H&RC) in Rangpur, recognized as the largest hypertension management center in the region and a leading national institute for hypertension research. A total of 2643 participants were enrolled from the center, with planned annual follow-ups until 2025. Each new patient visiting the center receives a unique patient identifier during their initial consultation. Baseline data were extracted from the H&RC record registry, and after applying the specified study criteria, information from 2276 patients was compiled at their first visit between January and March 2021. Details on the composition of the PREDIcT-HTN cohort can be found elsewhere [20]. Questionnaires administered during the initial follow-up visit covered blood pressure readings, behavioral factors, quality of life assessments, dietary habits, and medication adherence. Therefore, at the study's baseline, socio-demographic and clinical characteristics were documented for each participant, and antihypertensive medications were prescribed. Medication adherence, follow-up status and blood pressure control were evaluated during the initial follow-up visit.

### Study population and justification of sample size

The target population for the PREDIcT-HTN study comprises 5874 adult patients from northern Bangladesh who visited the H&RC between January and December 2020. Criteria for inclusion in the cohort were being at least 18 years old and prescribed medications for a confirmed hypertension. The final PREDIcT-HTN cohort included 2643 individuals. After the initial visit, 2276 patients remained in the baseline cohort, reflecting a non-response rate of 14%. For sample size calculation, we assumed that the group with poor treatment adherence would have an annual adverse event rate of less than 10%. Statistical calculations using the epi.cohort() function in the epiR package determined a sample size of 768 patients, providing sufficient power at a 5% significance level and 80% power to achieve the study's objectives [20, 21]. This current sample size of 2276 is expected to adequately address the study's goals.

### Outcome variable

Blood pressure (BP) was assessed thrice in the left arm, with a 3-min interval between each reading, following a minimum 10-min seated rest period before the initial measurement. In cases of significant deviation among the three readings, an additional measurement was conducted, and the recorded BP represented the average of the three closest values. Utilizing a cuff tailored to the arm's diameter and a mercury sphygmomanometer calibrated beforehand, BP measurements were taken. Patients' hypertensive conditions were classified into three categories: controlled BP, grade-I and grade-II uncontrolled hypertension, in accordance with the 2020 International Society of Hypertension Global Hypertension Practice Guideline [11]. In this study, BP control was assessed during the first follow-up visit.

### Medication adherence

The 14-item Hill-Bone Compliance to High Blood Pressure Therapy Scale (HB-HBP) was utilized to measure three crucial behavioral areas of treating high blood pressure: medication adherence (9 items), diet (2 items), and appointment keeping (3 items) [22]. Each item was rated on a 4-point Likert scale, with a score of 4 indicating the maximum level of compliance. The maximum and minimum scores for each of the 14 items were 56 and 14, respectively. The maximum possible score on the medication adherence subscale is 36. Higher ratings indicated worse adherence to antihypertensive medication. Three groups were created for the medication adherence variable from the scores of 9 items: scores > = 80% were termed as good, scores between 70–80% as moderate, and scores < 70% as low [23]. At baseline, patients were prescribed medications to manage hypertension, and their medication adherence was assessed during the first follow-up.

### Independent variables

Data were collected and categorized into two sets of independent predictors: socio-economic and clinical variables. Socio-economic variables comprised age (in years), which was further stratified into age groups: < = 30, 31–40, 41–50, 51–60, 61–70, and 70 + years, as well as sex (male and female). Additionally, socio-economic variables included schooling (no-schooling, 1–5, 6–10, 11–12 and 12 + years) and occupation (businessman, service-holder, day laborer, farmer, housewife and not working). Family income was recorded in Bangladeshi Taka (BDT) and categorized as: < = 10000, 10001–30000, and 30001–50000 + BDT. Residence status encompassed patients from rural areas, upazila, and cities.

Clinical variables were extracted from hospital records. The duration of hypertension diagnosis was categorized into three groups: new (< 2 years), 2–5 years, and > 5 years. Follow-up status was classified as regular or irregular. The number of comorbidities was categorized as follows: hypertension only, hypertension with one other disease, and hypertension with two or more other diseases. The number of medications prescribed to patients was classified as 1, 2, or > = 3. Body Mass Index (BMI), calculated from height and weight records, was categorized as normal (18.5–24.9 kg/m^2^), underweight (< 18.5 kg/m^2^), overweight (25–29.9 kg/m^2^), and obese (> = 30 kg/m^2^) (23).

### Statistical analysis

The questionnaires underwent rigorous scrutiny to ensure completeness, precision, and internal consistency, with any incomplete or inaccurate data excluded from analysis. Data analysis was performed using *R version 4.1.1 software* [24]. Categorical variables related to blood pressure control were presented as percentages and frequencies, while continuous variables were expressed as mean and standard deviation. The Chi-squared test was employed to detect significant differences between categorical variables. The potential confounding variables are evaluated using directed acyclic graph (DAG), which was followed by the multinomial regression model. The DAG was constructed using DAGITTY (http://www.dagitty.net/dags.html#), which helps to visualize and analyze relationships between variables. We also obtained variance inflation factors (VIF) in the logistic regression model to evaluate potential multicollinearity in the model (Supplement). Multinomial regression analyses were conducted to identify potential risk factors of uncontrolled hypertension status among adult hypertensive patients, with statistical significance set at p < 0.05 and a confidence interval of 95% for relative risk ratios (RRR). The RRR, derived from a multinomial logistic regression model, quantifies how the risk of uncontrolled hypertension (Grade-I and II) in a comparison group changes relative to the reference group with a one-unit change in the predictor variable, while keeping all other variables constant. The protocol, questionnaire, R scripts, and data are available at https://osf.io/sz7dt/.

## Results

### Characteristics of the participants

The mean age of 2276 hypertensive patients was 51.31 years (Standard Deviation: 11.58). Over half (56.1%) of the patients were aged between 40 and 60 years. The proportion of males and females was nearly equal. More than two-thirds (72.7%) of the patients resided in city or upazila areas (semi-urban), while approximately 27% were from rural areas. About 28% of patients had completed education beyond the higher secondary level, while 13.2% had no formal education. In terms of occupation, 42.2% of patients were housewives, and another 33% were employed in service roles. Only about 10% of respondents were engaged in agriculture or laborious work. Approximately half of the patients (49.3%) had a family income between 10,001 and 30,000 BDT. Additionally, roughly 21% fell into income category of less than 10,000 BDT.

**Fig. 1:**
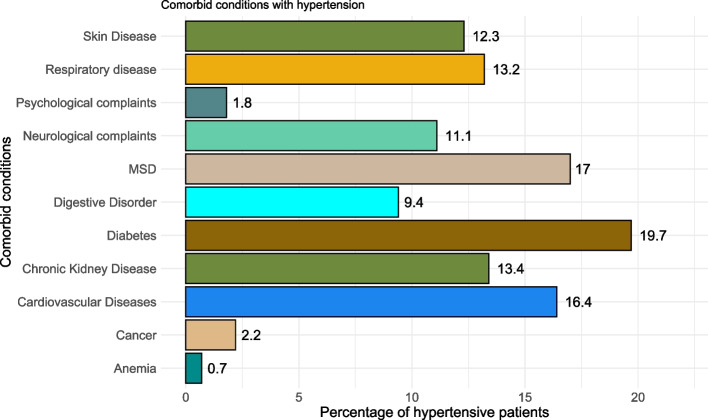
Comorbid conditions in hypertension

Figure 1 presents the comorbid conditions with hypertension. Approximately 20% of all hypertensive patients presented with coexistent diabetes, while musculoskeletal disorders exhibited the second highest prevalence at 17%, closely followed by cardiovascular diseases at 16.4%. Additionally, about 13% of patients manifested chronic kidney disease or respiratory diseases. Noteworthy proportions of diseases were also observed in neurological conditions (11.1%), skin diseases (12.3%), and digestive disorders (9.4%).

### Prevalence of uncontrolled BP in hypertensive participants

Around 60% of patients displayed uncontrolled BP, with 36.1% having grade-I hypertension and 24.2% with grade-II hypertension. Approximately 40% of patients maintained controlled or normal-range blood pressure. The average systolic and diastolic BPs were 140.5 ± 20.46 mmHg and 84.74 ± 11.31 mmHg, respectively. Table 1 outlines the distribution of BP control status among patients, categorized by socio-economic factors and clinical characteristics like BMI, duration of hypertension, medication adherence, follow-up patterns, and comorbidities.

**Table 1:** Prevalence of blood pressure control status among hypertensive adults

**Variables**	**Blood pressure control status**			**Total *n* = 2276 (%)**	*p*-value*
	**Controlled (*n* = 903, 39.7%)**	**Grade-I (*n* = 822, 36.1%)**	**Grade-II (*n* = 551, 24.2%)**		
**Sex**					
Male	449 (41.2)	376 (34.5)	265 (24.3)	1090 (47.9)	0.253
Female	454 (38.3)	446 (37.6)	286 (24.1)	1186 (52.1)	
**Age-group (in years) [Mean age = 51.31 Years standard deviation = 11.58 years]**					
≤ 30	25 (39.1)	22 (34.4)	17 (26.6)	64 (2.8)	**< 0.001**
31–40	154 (42.1)	142 (38.8)	70 (19.1)	366 (16.1)	
41–50	282 (44.3)	224 (35.2)	130 (20.4)	636 (28.0)	
51–60	250 (39.1)	229 (35.8)	160 (25.0)	639 (28.1)	
61–70	159 (36.1)	159 (36.1)	123 (27.9)	441 (19.4)	
70 +	32 (25.2)	36 (36.2)	49 (38.6)	127 (5.6)	
**Occupation**					
Business	105 (36.7)	105 (36.7)	76 (26.6)	286 (12.6)	**< 0.001**
Day Labor	18 (40.0)	19 (42.2)	8 (17.8)	45 (2.0)	
Farmer	61 (35.1)	54 (31.0)	59 (33.9)	174 (7.6)	
Housewife	364 (37.9)	347 (36.1)	250 (26.0)	961 (42.2)	
Not working	21 (30.9)	26 (38.2)	21 (30.9)	68 (3.0)	
Service	333 (44.9)	271 (36.5)	138 (18.6)	742 (32.6)	
**Residence**					
Rural	226 (36.5)	217 (35.0)	177 (28.5)	620 (27.2)	0.165
Upazila (semi-urban)	317 (40.3)	309 (39.3)	160 (20.4)	786 (34.5)	
City	360 (41.4)	296 (34.0)	214 (24.6)	870 (38.2)	
**Schooling**					
No-schooling	97 (32.2)	105 (34.9)	99 (32.9)	301 (13.2)	**0.004**
1–5 years	142 (33.7)	161 (38.2)	118 (28.0)	421 (18.5)	
6–10 years	237 (39.2)	232 (38.4)	135 (22.4)	604 (26.5)	
11–12 years	144 (45.6)	106 (33.5)	66 (20.9)	316 (13.9)	
12 + years	283 (43.6)	218 (34.4)	133 (21.0)	634 (27.9)	
**Family Income**					
≤ 10000 BDT	184 (38.3)	164 (34.2)	132 (27.5)	480 (21.1)	0.166
10001–30000 BDT	451 (40.2)	395 (35.2)	275 (24.5)	1121 (49.3)	
30001–50000 BDT	192 (38.6)	192 (38.6)	114 (22.9)	498 (21.9)	
50000 + BDT	76 (42.9)	71 (40.1)	30 (16.9)	177 (7.8)	
**BMI Category**					
Underweight	29 (48.3)	17 (28.3)	14 (23.3)	60 (2.6)	**0.045**
Normal weight	381 (37.1)	369 (35.9)	277 (27.0)	1027 (45.1)	
Overweight	367 (42.4)	312 (36.1)	186 (21.5)	865 (38.1)	
Obese	125 (38.7)	124 (38.4)	74 (22.9)	323 (14.2)	
**Duration of Hypertension**					
> 5 years	305 (41.2)	278 (37.5)	158 (21.3)	741 (33.1)	**0.028**
2–5 years	404 (40.4)	354 (35.4)	242 (24.2)	1012 (45.2)	
New patient	175 (35.4)	176 (35.6)	144 (29.1)	504 (22.5)	
**Follow-up Status**					
Regular	677 (43.4)	582 (37.3)	301 (19.3)	1560 (68.5)	**< 0.001**
Irregular	226 (31.6)	240 (33.5)	234 (34.9)	716 (31.5)	
**Comorbid conditions in hypertension**					
Only hypertensive	435 (39.8)	398 (36.4)	260 (23.8)	1093 (48.0)	**0.031**
Hypertensive patients with one comorbid disease	315 (43.0)	254 (34.7)	164 (22.4)	733 (32.2)	
Hypertensive patients with two or more comorbid diseases	153 (34.0)	170 (37.8)	127 (28.2)	450 (19.8)	
**Number of prescribed medications per day**					
1	606 (41.4)	532 (36.3)	327 (22.3)	1465 (64.4)	**< 0.001**
2	286 (37.9)	274 (36.3)	194 (25.7)	754 (33.1)	
> = 3	11 (19.3)	16 (28.1)	30 (52.6)	57 (2.5)	
**Medication Adherence**					
Poor Adherence	97 (28.9)	126 (37.3)	113 (33.6)	336 (14.8)	**< 0.001**
Moderate Adherence	739 (41.6)	641 (36.1)	395 (22.3)	1175 (78.0)	
Good Adherence	67 (40.6)	55 (33.3)	43 (26.1)	165 (7.2)	

^*^p-values are from chi-squared test and bold faces represents significant at 5% significance level

Variations in the prevalence of uncontrolled BP were noted across different age groups, with a higher prevalence of Grade-II hypertension as age increased. For instance, the prevalence of grade-II hypertension was 26.6% for those under 30 years and increased to 38.6% for individuals aged 70 years and above. Among different employment categories, farmers (33.9%), housewives (26%), and unemployed individuals (30.9%) showed higher rates of grade-II hypertension.

Education appeared to play a role in effective hypertension control, as the prevalence of uncontrolled hypertension was highest among patients with no schooling (32.9%) and lowest among those with over 12 years of education (21%).

Among patients with varying BMI categories, the prevalence of uncontrolled hypertension was consistent across underweight (23.3%), overweight (21.5%), and obese (22.9%) individuals, while the highest prevalence of grade-II hypertension was observed in the normal weight category (27%). Newly diagnosed hypertensive patients (29.1%) had a higher rate of grade-II hypertension compared to those diagnosed 2–5 years earlier (24.6%). Notably, lower levels of medication adherence were associated with a higher proportion of uncontrolled blood pressure. Poor medication adherence was observed in approximately 15% of patients, with 7.2% demonstrating good adherence. Patients with poor adherence exhibited a higher prevalence of grade-II hypertension (33.6%) compared to those with good adherence (26.1%). Irregular follow-up records were linked to a higher proportion of grade-II BP (34.9%) compared to regular follow-up (19.3%). Furthermore, among patients with comorbidities, the highest percentage of uncontrolled BP (52.6%) was observed in those with more than two comorbidities.

### Level of medication adherence among the hypertensive patients

Medication adherence was evaluated using the 9-item Hill-Bone medication adherence scale, categorizing patients into poor, moderate, and good adherence levels. The majority of patients (78%) demonstrated moderate adherence, while approximately 15% exhibited poor adherence, and only 7% showed good adherence. Table 2 provides a breakdown of medication adherence levels across various sociodemographic and clinical factors.

**Table 2:** Prevalence of medication adherence to anti-hypertensive drugs

**Variable**	**Medication Adherence**				*p*-value*
	**Poor (*n* = 336, 14.76%)**	**Moderate (*n* = 1775, 77.98%)**	**Good (*n* = 165, 7.25%)**	**Total (*n* = 2276, 100%)**	
**Sex**					
Male	133 (12.2)	873 (80.1)	84 (7.7)	1090 (47.9)	**0.004**
Female	203 (17.1)	902 (76.1)	81 (6.8)	1186 (52.1)	
**Age-group (in years)**					
≤ 30	9 (14.1)	52 (81.2)	3 (4.7)	64 (2.8)	**0.072**
31–40	46 (12.6)	305 (83.3)	15 (4.1)	366 (16.1)	
41–50	84 (13.2)	496 (78.0)	56 (8.8)	636 (28.0)	
51–60	112 (17.5)	480 (75.1)	47 (7.4)	639 (28.1)	
61–70	61 (13.8)	346 (78.5)	34 (7.7)	441 (19.4)	
70 +	23 (18.1)	94 (74.0)	10 (7.9)	127 (5.6)	
**Occupation**					
Business	39 (13.6)	227 (79.4)	20 (7.0)	286 (12.6)	**< 0.001**
Day Labor	7 (15.6)	36 (80.0)	2 (4.4)	45 (2.0)	
Farmer	38 (21.8)	115 (66.1)	21 (12.1)	174 (7.6)	
Housewife	165 (17.2)	727 (75.7)	69 (7.2)	961 (42.2)	
Not working	9 (13.2)	54 (79.4)	5 (7.4)	68 (3.0)	
Service	78 (10.5)	61.6 (83.0)	48 (6.5)	742 (32.6)	
**Residence**					
Rural	139 (22.4)	407 (65.6)	74 (11.9)	620 (27.2)	**< 0.001**
Upazila (semi-urban)	86 (10.9)	671 (85.4)	29 (3.7)	786 (34.5)	
City	111 (12.8)	697 (80.1)	62 (7.1)	870 (38.2)	
**Schooling**					
No schooling	65 (21.6)	211 (70.1)	25 (8.3)	301 (13.2)	**< 0.001**
1–5 years	78 (18.5)	311 (73.9)	32 (7.6)	421 (18.5)	
6–10 years	92 (15.2)	462 (76.5)	50 (8.3)	604 (26.5)	
11–12 years	39 (12.3)	258 (81.6)	19 (6.0)	316 (13.9)	
12 + years	62 (9.8)	533 (84.1)	39 (6.2)	634 (27.9)	
**Family Income (BDT)**					
≤ 10,000	102 (21.2)	318 (66.2)	60 (12.5)	480 (21.1)	**< 0.001**
10001–30000	154 (13.7)	902 (80.5)	65 (5.8)	1121 (49.3)	
30001–50000	64 (12.9)	402 (80.7)	32 (6.4)	498 (21.9)	
50000 +	16 (9.0)	153 (86.4)	8 (4.5)	177 (7.8)	
**BMI Category**					
Normal weight	158 (15.4)	799 (77.8)	70 (6.8)	1027 (45.1)	0.169
Underweight	16 (26.7)	40 (66.7)	4 (6.7)	60 (2.6)	
Overweight	117 (13.5)	684 (79.1)	64 (7.4)	865 (38.0)	
Obese	45 (13.9)	251 (77.7)	27 (8.4)	323 (14.2)	
**Duration of Hypertension**					
> 5 years	115 (15.5)	556 (77.2)	54 (7.3)	741 (33.1)	**0.033**
2–5 years	126 (12.6)	810 (81.0)	64 (6.4)	1000 (44.7)	
New	88 (17.8)	366 (73.9)	41 (8.3)	495 (22.1)	
**Follow-up Status**					
Regular	156 (9.8)	1327 (83.5)	107 (6.7)	1590 (69.9)	**< 0.001**
Irregular	180 (26.2)	448 (65.3)	58 (8.5)	686 (30.1)	
**Comorbid conditions in hypertension**					
Only hypertensive	148 (13.5)	872 (79.8)	73 (6.7)	1093 (48.0)	**0.043**
Hypertensive patients with one comorbid disease	115 (15.7)	552 (75.3)	66 (9.0)	733 (32.2)	
Hypertensive patients with two or more comorbid diseases	73 (16.2)	331 (73.5)	46 (10.2)	450 (19.8)	
**Number of prescribed medications per day**					
1	198 (13.5)	1170 (79.9)	97 (6.6)	1465 (64.4)	**0.029**
2	132 (17.5)	560 (74.3)	62 (8.2)	754 (33.1)	
> = 3	6 (10.5)	45 (78.9)	6 (10.5)	57 (2.5)	

^*^p-values are from chi-squared test and bold faces represents significant at 5% significance level

Females (17.1%) had higher rates of poor adherence than males (12.2%). Patients over 70 years old having the highest percentage of poor adherence (18.1%) among age groups. Conversely, good adherence was less prevalent among those aged < = 30 (4.7%) and 31–40 (4.1%). Farmers (21.8%) and housewives (17.2%) showed higher rates of poor adherence compared to other occupations. Rural residents (22.4%) had higher rates compared to city dwellers (12.2%). Lower education levels were linked to higher rates of poor adherence, with patients having no schooling (21.6%) or primary education (18.5%) showing elevated proportions of poor adherence. Similarly, patients with lower family income exhibited higher rates of poor medication adherence, with those earning less than 10,000 BDT having double the proportion of poor adherence compared to those earning more than 50,000 BDT.

Furthermore, the prevalence of poor medication adherence increased with the number of comorbidities. Patients with more than two comorbid conditions (16.2%) had higher rates poor adherence than those without comorbid conditions (13.5%. Patients with irregular follow-up records had a threefold higher proportion of poor medication adherence (26.2%) compared to those with regular follow-up (9.8%). Newly diagnosed hypertensive patients (17.2%) had higher rates poor adherence than those diagnosed 2–5 years earlier (12.6%). Additionally, underweight patients had the highest prevalence of poor adherence (26.7%) among BMI categories.

### Potential risk factors of uncontrolled blood pressure

The Directed Acyclic Graph (DAG) was created to evaluate confounder selection in the multinomial regression model (Appendix). Table 3 presents the results of the multinomial regression analysis, considering potential confounders such as age, sex, residence, duration of hypertension, and number of comorbid conditions when investigating the relationship between medication adherence and blood pressure control status.

**Table 3:** Associated factors for uncontrolled hypertension (Grade-I and II) of the hypertensive patients: Multinomial logistic regression analysis

**Variables**	**Grade-I versus Controlled**		**Grade-II versus Controlled**	
	**RRR**	**95% CI**	**RRR**	**95% CI**
**Sex (Ref: Male)**				
Female	1.19	0.98–1.44	1.08	0.87–1.35
**Age-group (Ref: < = 30 years)**				
31–40	1.14	0.60–2.15	0.72	0.35–1.45
41–50	0.97	0.52–1.81	0.76	0.39–1.51
51–60	1.13	0.60–2.12	1.10	0.56–2.17
61–70	1.23	0.64–2.34	1.40	0.70–2.82
70 +	1.75	0.82–3.75	**2.72**	**1.22–5.95**
**Duration of Hypertension (Ref: New patient)**				
2–5	0.87	0.68–1.13	**0.75**	**0.57–0.99**
> 5	0.86	0.65–1.13	**0.53**	**0.39–0.73**
**Residence (Ref: Rural)**				
Upzila (Semi-urban)	1.04	0.81–1.34	**0.72**	**0.54–0.97**
City	0.89	0.70–1.14	0.86	0.65–1.11
**Comorbid conditions in hypertension (Ref: No coexisted disease)**				
Hypertensive patients with one comorbid disease	0.86	0.69–1.07	**0.79**	**0.60–1.00**
Hypertension and presence of ≥ 2 diseases	1.13	0.86–1.47	1.22	0.91–1.65
**Medication Adherence (Ref: Poor)**				
Moderate	0.70	0.52–0.93	**0.50**	**0.36–0.68**
Good	0.68	0.43–1.07	**0.56**	**0.35–0.91**

The analysis reveals that the risk of severe hypertension increases substantially with age. Older adults, particularly those aged 60 years and above, are at high risk of developing grade-II hypertension. For instance, patients over 70 years of age were about 2.72 times more likely to develop grade-II hypertension compared to those under 30 years (RRR: 2.72, 95% CI: 1.22–5.95). Interestingly, younger males (under 30 years) were more prone to have grade-II hypertension compared to younger females (Appendix).

Patients diagnosed with hypertension 2–5 years earlier had a 25% lower risk of developing grade-II hypertension compared to newly diagnosed patients (RRR = 0.75, 95% CI = 0.57–0.99), while those diagnosed more than 5 years earlier had a 47% lower risk (RRR = 0.53, 95% CI = 0.39–0.73). Poor medication adherence, adjusted for age, sex, residence, or comorbidities, emerged as a significant risk factor for uncontrolled hypertension. Individuals with good medication adherence had a 44% lower risk of developing grade-II hypertension compared to those with poor adherence (RRR = 0.56, 95% CI = 0.35–0.91).

The results highlight a complex interplay between medication adherence, age, follow-up visits, comorbid conditions, and the development of severe hypertension. The presence of other health conditions can worsen hypertension. Patients with more than two comorbid conditions had a similar risk of developing grade-II hypertension as those with hypertension only. However, having only one comorbid condition compared to hypertension alone reduced the risk of severe hypertension by 21% (RRR = 0.79, 95% CI = 0.60–1.00).

While age is a well-established risk factor for worsening hypertension, our study explored other contributing factors, detailed in the supplementary figures. The analysis revealed a critical trend: consistent medication adherence, regular doctor follow-ups, and managing coexisting health conditions (comorbidities) significantly prevent the progression to severe hypertension. This finding emphasizes the need for targeted interventions to improve medication adherence, particularly among high-risk groups. These groups include older adults, those living in rural areas, and individuals with additional health conditions.

## Discussion

This study in Bangladesh is the first to use a baseline and first follow-up data from a prospective cohort design to assess the impact of medication adherence, follow-up visits, comorbidities, and sociodemographic factors on blood pressure control in hypertensive patients. Baseline data was collected, and patients were followed over time. Alarmingly, the study showed that more than 6 in 10 patients struggled to control their blood pressure. Within this group, over a third had a grade-I elevation, and about a quarter had a more concerning grade-II elevation. A 2023 World Health Organization (WHO) report estimated that globally, only about one-fourth of adults with hypertension have their blood pressure controlled [25]. Another study conducted in rural South Asia, focusing Bangladesh, Pakistan, and Sri Lanka, revealed a high prevalence of uncontrolled blood pressure among hypertensive patients, with approximately 58% of patients across all three countries struggling to manage their blood pressure effectively [26, 27].

In our study, it was observed that approximately 78% of patients exhibited moderate medication adherence, while 15% showed poor medication adherence. A meta-analysis indicated that medication nonadherence to antihypertensive medications may be more prevalent in African and Asian populations, with an estimated prevalence of around 62.5% [28]. Conversely, patients with moderate to good medication adherence levels had a notably lower risk of uncontrolled BP compared to those with low adherence. These findings align with a hospital-based cross-sectional study in Bangladesh, where 85.9% of patients with medication non-adherence were found to have uncontrolled hypertension [29]. Similar associations between low medication adherence and uncontrolled BP have been observed in studies conducted in Bangladesh, India, Pakistan, and Sri Lanka [30, 31]. Studies from various global regions have also highlighted the impact of suboptimal medication adherence on poor BP control among individuals with hypertension [26, 27, 30].

Our findings further revealed that patients with irregular follow-up had an elevated risk of progressing to grade-II hypertension compared to those with regular follow-up. This observation is consistent with studies conducted on hypertensive patients in Pakistan, China, and Turkey, which reported an association between low attendance at follow-up visits and uncontrolled BP among participants [32–37]. Skipping appointments can lead to missed opportunities to adjust medications or address issues that might be contributing to uncontrolled BP.

Furthermore, our observations indicated that older adults faced an increased risk of developing grade-II hypertension. This correlation is consistent with results from a population-based survey in Bangladesh and various cross-sectional studies in Thailand and Ethiopia, all of which identified advancing age as a predictor of uncontrolled hypertension [8, 38, 39]. Age was found to play a crucial role in influencing both medication adherence and hypertension management, underscoring the necessity for customized interventions aimed at different age demographics to enhance treatment effectiveness. Older adults might face challenges with medication adherence due to factors like memory issues, polypharmacy (taking multiple medications), or difficulty with medication routines.

In our research, we observed that newly diagnosed hypertensive patients were at a greater risk of advancing to grade-II hypertension compared to those diagnosed over five years ago. This aligns with a study in Ethiopia, which reported a reduced risk of uncontrolled hypertension among patients diagnosed five to ten years earlier [40]. The heightened risk for newly diagnosed patients likely have a higher initial risk due to the absence of consistent medication control.

In addition to evaluating the impact of medication adherence on BP control, this study identified specific socio-demographic groups with a high incidence of poor medication adherence. Particularly, low medication adherence was noted among individuals with lower levels of education and family income. This discovery is in line with prior research conducted by Hussain et al., whose cross-sectional study in Bangladesh identified low education and income as factors associated with medication non-adherence [41]. Similar trends were observed in other cross-sectional studies in Bangladesh and Pakistan, emphasizing low education as a contributing factor to inadequate medication adherence [34, 42].

Moreover, our study revealed a high prevalence of poor medication adherence among patients in rural areas, consistent with previous research in healthcare settings in Bangladesh and Pakistan [29, 42]. Notably, a study on antihypertensive medication adherence in rural Bangladesh also highlighted a significant proportion of participants exhibiting non-adherence to medications [34]. Individuals residing in rural areas may encounter difficulties such as self-discontinuation of medication without medical consultation, adjusting dosages independently, or forgetting to adhere to prescribed medication regimens. Moreover, rural regions frequently experience a lack of sufficient healthcare infrastructure and educational initiatives, which can contribute to decreased medication adherence. Consequently, patients in rural settings often demonstrate lower levels of adherence, resulting in challenges in controlling hypertension effectively.

Furthermore, our analysis uncovered a significantly higher prevalence of poor medication adherence among patients with more than two comorbidities. This finding aligns with results from a cross-sectional study in Saudi Arabia, which reported a higher incidence of medication non-adherence in patients with one or more comorbidities [43]. The presence of multiple comorbidities can exacerbate the challenges in controlling blood pressure effectively. The complex task of managing multiple medications, often seen in hypertensive patients with comorbidities, can negatively impact adherence to medication regimens, ultimately hindering successful blood pressure control.

### Limitations and strengths

There are a few strengths and limitations that should be considered when interpreting the study's findings and their applicability to broader contexts. While baseline data is crucial in cohort studies for understanding the initial characteristics of the group, its limitations should be acknowledged. The baseline analysis might not definitively prove that a factor measured at baseline caused a later outcome. Although the study encompassed patients from northern Bangladesh, its applicability to the broader population of Bangladesh is plausible due to similar demographics. However, the findings may not be broadly applicable to populations with distinct demographics, healthcare systems, or cultural influences impacting medication adherence. It is important to note that medication adherence assessment in the study may have relied on self-reported data, which could introduce bias. Utilizing a dose–response analysis approach with outcome measurements at intervals could have strengthened the evidence by accounting for the varying timeframes between admission and outcome assessment among participants. While patients were thoroughly evaluated and treated for chronic conditions like diabetes, hypertension, cardiovascular disease, and respiratory illnesses, the focus on these diseases may have contributed to insufficient identification and documentation of other comorbidities, thereby affecting the comprehensiveness of the overall health assessments.

The study placed a strong emphasis on medication adherence, a critical factor often under-addressed in hypertension management research. By identifying a high-risk group susceptible to both uncontrolled hypertension and medication adherence challenges, the study paved the way for targeted interventions. With a large sample size, the study's findings are more likely to be generalizable. Patient selection from a prominent national hypertension management center catering to individuals from diverse sociodemographic backgrounds, encompassing significant geographical and economic diversity, further enhances the study's generalizability. Additionally, comprehensive data collection on various sociodemographic and health-related factors, coupled with adjustments for confounding effects during analysis, bolstered the validity and relevance of the study results.

## Conclusion

The high prevalence of uncontrolled hypertension in Bangladesh poses a significant public health concern. This study underscores the critical role of medication adherence, regular follow-up visits, and effective management of comorbid conditions in maintaining BP control among hypertensive patients in Bangladesh. The management of hypertension necessitates a comprehensive approach that considers comorbidities, medication adherence, and sociodemographic factors. The study's findings offer crucial guidance for national healthcare policymakers to enhance hypertension management strategies, emphasizing the importance of addressing medication adherence and follow-up practices, while considering variations in age groups and patient residence (rural vs. urban). This evidence can inform the development of a comprehensive hypertension management strategy, advancing the national non-communicable disease prevention and control agenda in alignment with the Sustainable Development Goals by 2030.

## Supplementary Information

Supplementary Material 1.

Supplementary Material 2.

## Data Availability

The de-identified dataset, protocol, ethical approval letter from NSU and questionnaire can be found in the source https://osf.io/sz7dt/.
