# Supplementary material for: Medication adherence and blood pressure control in treated hypertensive patients: first follow-up findings from the PREDIcT-HTN study in Northern Bangladesh
Source: BMC Public Health. 2025 Jan 21;25:250. doi: 10.1186/s12889-025-21409-z (PMC11748311; doi:10.1186/s12889-025-21409-z)
Supplement: Supplementary file 1 — Supplementary Material 1. [file 12889_2025_21409_MOESM1_ESM.docx]

**Title: Medication Adherence and Blood Pressure Control in Treated Hypertensive Patients: Baseline Findings from the PREDIcT-HTN Study in Northern Bangladesh**

Table of Contents

[Appendix A1: Logistic regression: Grade I versus controlled hypertension 2](#_Toc167100930)

[Appendix A2: Logistic regression: Grade II versus controlled hypertension 3](#_Toc167100931)

[Appendix A3: Directed Acyclic Graph (DAG) 4](#_Toc167100932)

[Appendix A4: Trend analysis by age and grade II hypertension 5](#_Toc167100933)

# Appendix A1: Logistic regression: Grade I versus controlled hypertension


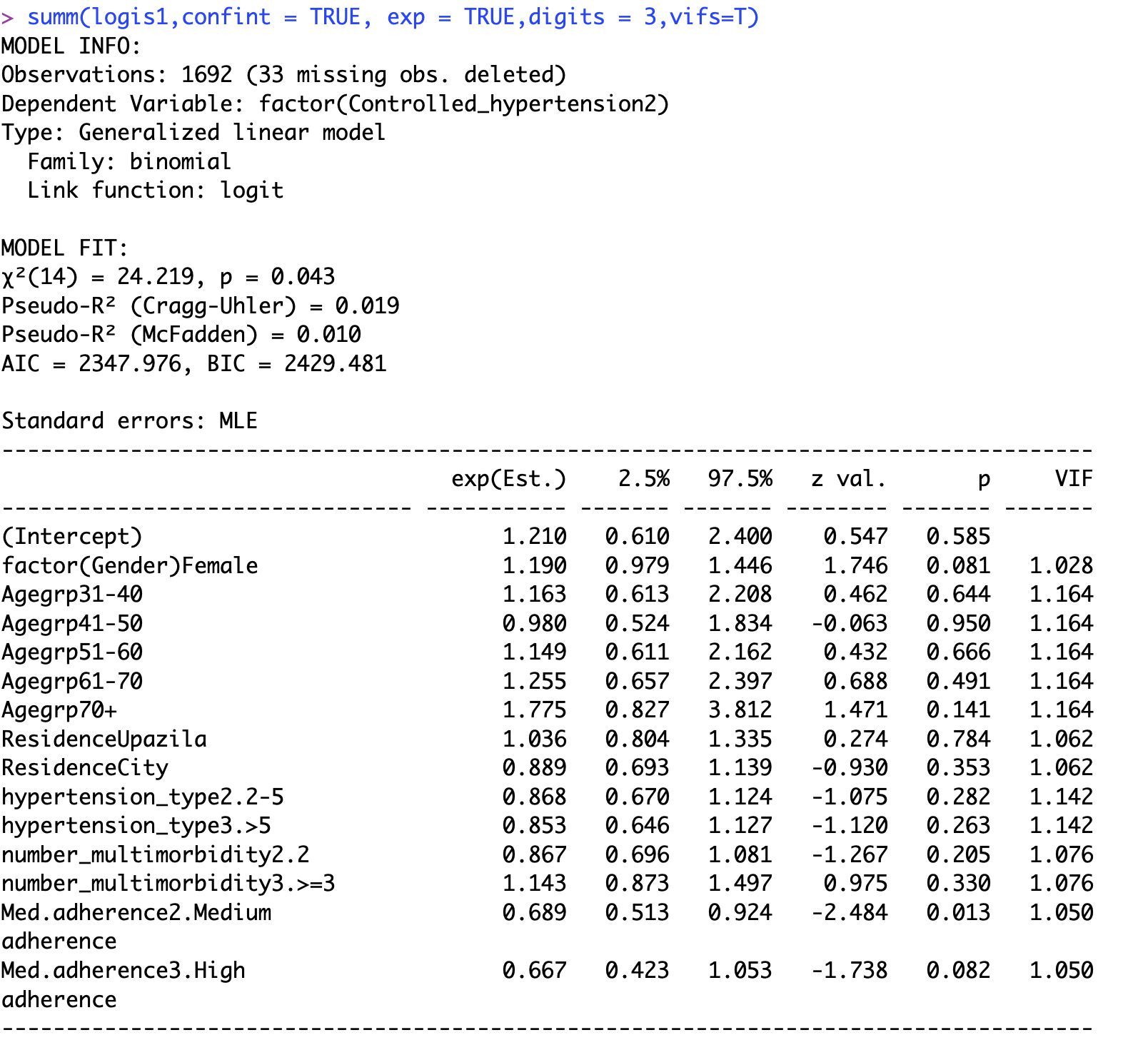


The VIF is close to 1, which indicates no correlation between the covariate (independent variable) and other independent variables in the model. There's no inflation of the variance of the coefficient estimate due to multicollinearity.

# Appendix A2: Logistic regression: Grade II versus controlled hypertension


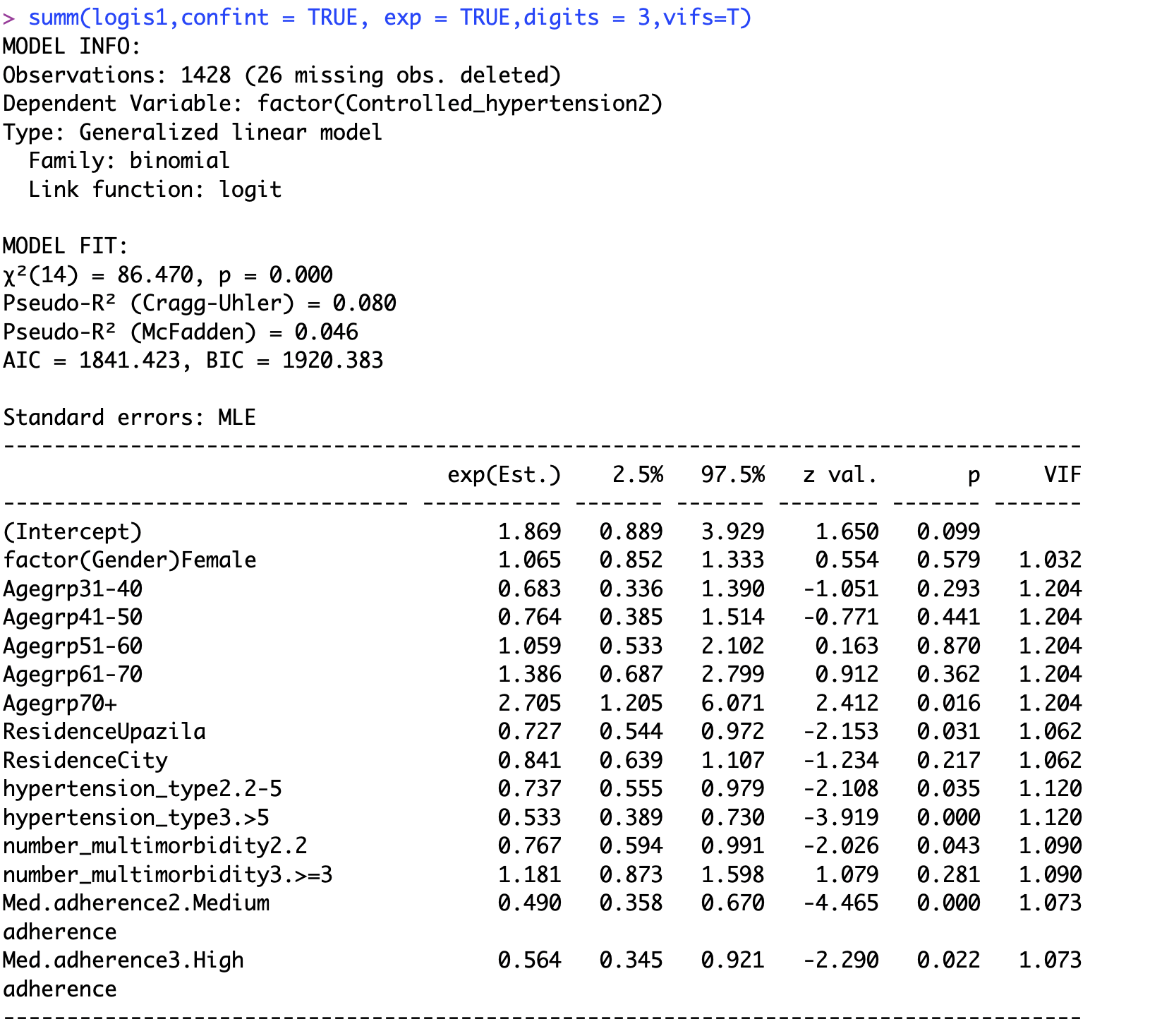


The VIF is close to 1, which indicates no correlation between the covariate (independent variable) and other independent variables in the model. There's no inflation of the variance of the coefficient estimate due to multicollinearity.

# Appendix A3: Directed Acyclic Graph (DAG)


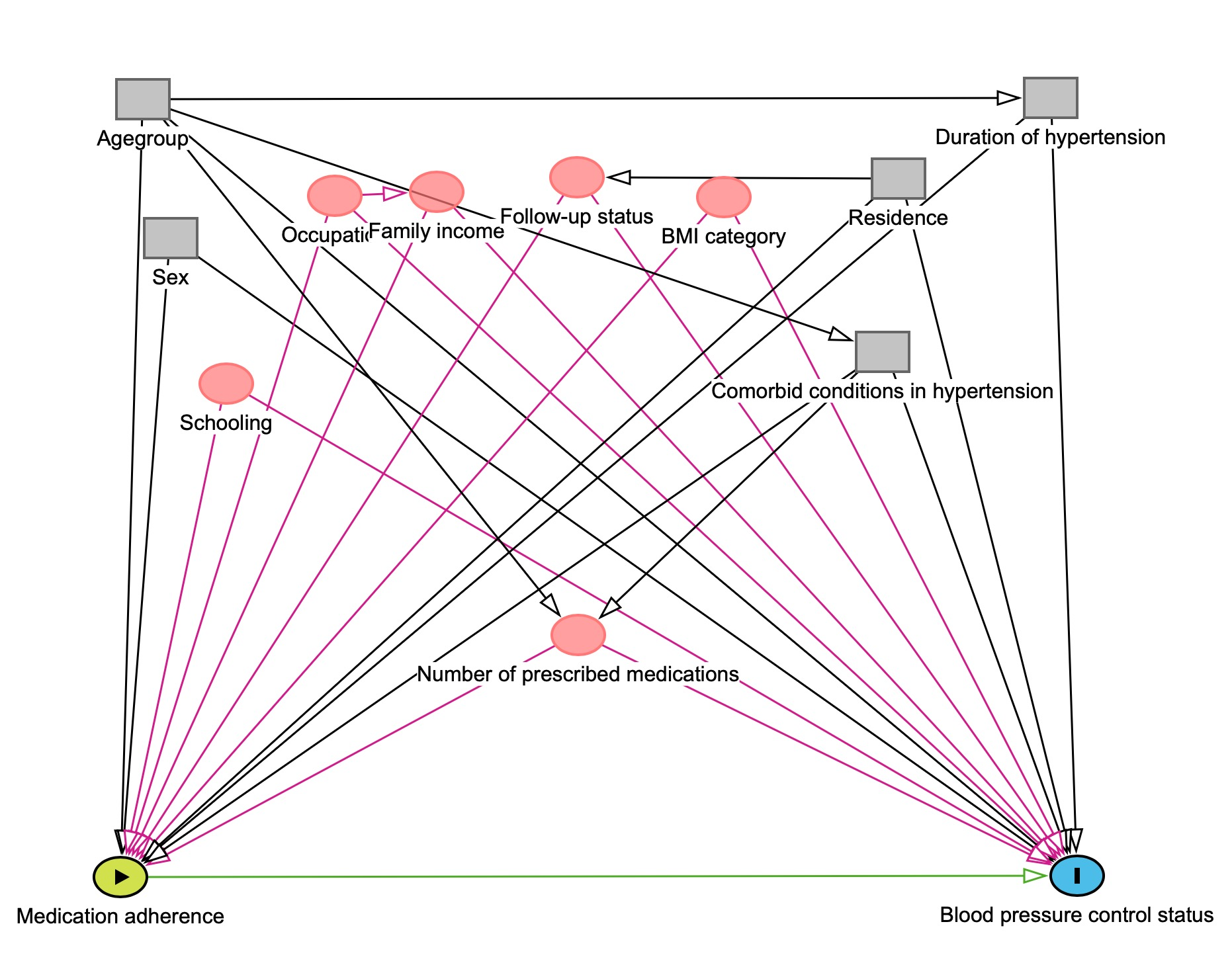


This Directed Acyclic Graph (DAG) illustrates the evaluation of covariate selection in analyzing the effects of medication adherence on blood pressure control status. In this diagram, medication adherence serves as the exposure variable, while blood pressure control status is depicted as the outcome variable. Nodes highlighted in green with a black border represent the exposure variable, and those in blue with a black border represent the outcome variable. The blue nodes signify ancestors of the outcome variable. Green nodes represent ancestor of the exposure, while red nodes denote potential confounders. The red paths within the diagram indicate the biasing paths. The green paths represent the causal paths under consideration. In this instance, all the other exposures (‘nodes’) are theoretically causally associated with (i.e., ancestors of) both the exposure and the outcome. To adjust for confounding in the association of interest, it is necessary to close all ‘backdoor pathways’ between the exposure and outcome (i.e., any pathway (consisting of a series of one or more edges and nodes) that provides an alternate route between the exposure and outcome); this is accomplished by adjusting for at least one node on that path. The minimally sufficient adjustment set is the combination of the fewest nodes that, being ancestors of both the exposure and outcome, if selected, effectively block all backdoor pathways between the exposure and the outcome (white nodes with black borders). These ‘adjusted variables’ are then introduced into the multivariate modelling as potential confounders. This figure is constructed through DAGitty (<http://www.dagitty.net> ).

# Appendix A4: Trend analysis by age and grade II hypertension

|  |  |
| --- | --- |
|  |  |
|  |  |

Figure: Trend analysis by age (well established risk factor) and other potential risk factors.
